# Differential Susceptibility May Not Drive Chytridiomycosis Related Declines in Multi‐Host Amphibian Communities

**DOI:** 10.1002/ece3.73201

**Published:** 2026-03-08

**Authors:** Elise Ringwaldt, Shannon Troy, Annie Philips, Scott Carver

**Affiliations:** ^1^ School of Natural Sciences University of Tasmania Hobart Tasmania Australia; ^2^ The Department of Natural Resources and Environment Tasmanian Government Hobart Tasmania Australia; ^3^ Odem School of Ecology University of Georgia Athens Georgia USA

**Keywords:** amphibian, *Batrachochytrium dendrobatidis*, chytridiomycosis, community, multi‐host disease, temperate species

## Abstract

*Batrachochytrium dendrobatidis* (Bd) is the most catastrophic wildlife pathogen, associated with severe amphibian population declines or the extinction of over 500 species. Bd has the potential to influence the structure and dynamics of amphibian populations in multiple and compounding ways, yet few studies have investigated how Bd effects vary in communities with differential susceptibility. Here, we used temporal data from amphibian communities in temperate Tasmania, Australia, comprising four co‐occurring species: the brown treefrog ( *Litoria ewingii* ), Tasmanian treefrog ( *L. burrowsae* ), common froglet ( *Crinia signifera* ), and Tasmanian froglet ( *C. tasmaniensis* ). Previous laboratory trials indicated *L. burrowsae* and *C. tasmaniensis* are susceptible to Bd, whereas *L. ewingii* and *C. signifera* act as reservoirs. Using Bayesian Markov chain Monte Carlo models, we tested whether Bd presence, the presence of reservoirs, or same‐genus species influenced susceptible species across sites and years. Contrary to expectations—that Bd and reservoir hosts destabilise amphibian communities—we found no evidence of population declines in *L. burrowsae* or *C. tasmaniensis* . Instead, species appeared to coexist in a relatively stable community structure, despite Bd presence. Our findings suggest that susceptibility identified by laboratory trials might not reliably predict field disease outcomes in this system, owing to an absence of relevant extrinsic environmental variables. We postulate that pond conditions in which amphibian communities in our study occur, characterised by low temperature and pH, limit Bd growth and survival, creating a refuge from its effects. These results highlight the importance of ecological and environmental context when assessing disease impacts in wild multi‐host communities, and also that climate change may threaten community resilience.

## Graphical abstract

We investigated how *Batrachochytrium dendrobatidis* (Bd) affects multi‐host amphibian communities in Tasmania, Australia. Despite differential susceptibility, we found no evidence of population declines in susceptible species, suggesting that current environmental conditions may buffer communities against Bd impacts. Our findings highlight the importance of ecological context when assessing wildlife disease outcomes.

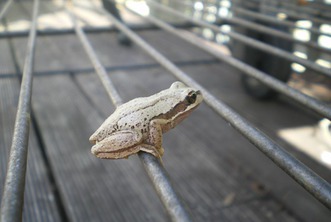

## 1 Introduction

Multi‐host diseases have the potential to influence the structure and dynamics of communities in multiple and compounding ways. This has been demonstrated in avian malaria (*Plasmodium relictum*) in Hawaiian birds (Warner 1968; Van Riper et al. 1986), canine distemper virus in carnivores (Roelke‐Parker et al. 1996; Loots et al. 2017), and bat communities infected with white nose syndrome (Blehert et al. 2009; Hoyt et al. 2021). Indeed, the extent to which multi‐host pathogens can affect community composition has increasingly been recognised (LaDeau et al. 2007; Johnson et al. 2015; Zipkin et al. 2020; Stewart Merrill and Johnson 2025). However, most research on multihost pathogens in wildlife has focused on one or few host species, rather than considering the range of reservoirs and incidental hosts within a community. This likely reflects the challenges of disentangling disease dynamics in wild systems, where environmental factors, host identity, and their interactions can amplify or dampen pathogen impacts (Dobson 2004; Hoyt et al. 2021; Hawley and Altizer 2011; Blaustein et al. 2012; Dunn et al. 2012; Murray et al. 2013; Cohen et al. 2017). More recently, research has begun to move beyond focal species to examine how host assemblages shape pathogen transmission and persistence (Caron et al. 2015; Ferreira et al. 2024; Daversa et al. 2022, 2025). Because emerging infectious diseases of wildlife can drive biodiversity loss, investigating their dynamics within a community structure is crucial for conservation planning and mitigating extinction risks (Daszak et al. 2000; Johnson et al. 2015).

One of the most catastrophic wildlife pathogens is *Batrachochytrium dendrobatidis* (hereafter Bd). Bd causes chytridiomycosis in amphibians and is associated with population declines and extinction, impacting over 500 amphibian species and devastating communities globally (Berger et al. 1998; Lips et al. 2006; Skerratt et al. 2007; Scheele et al. 2019). Mass‐mortality events owing to chytridiomycosis date back to the late 1970s (Berger et al. 1998; Lips et al. 2006; Voyles et al. 2009). Field and laboratory studies indicate that Bd is capable of infecting up to 54% of amphibian species (Castro Monzon et al. 2020), and is driving declines through a variety of mechanisms. Following invasion of Bd at a site, the pathogen can become enzootic and continue to cause mortality for years (Murray et al. 2009; Phillott et al. 2013). Adding complexity, the susceptibility of the host to the disease can be further influenced by environmental conditions (Piotrowski et al. 2004; Nowakowski et al. 2016; Cohen et al. 2017; Sonn et al. 2020).

Since its discovery, a substantive body of literature on Bd‐driven amphibian declines has developed. The overwhelming majority of studies have focused on single species and their susceptibility to infection (Skerratt et al. 2016; Scheele, Skerratt, et al. 2017), and surprisingly few have considered interactions between species within wild communities. Although many studies have focused on two‐species interactions, typically between a reservoir and a susceptible host (Parris and Beaudoin 2004; Bosch and Rincón 2008), assemblage‐level impacts of Bd are increasingly being explored (DiRenzo et al. 2017; Gervasi et al. 2013; Brannelly, Webb, et al. 2018; Brannelly, Clemann, et al. 2018; Bielby et al. 2021; Longo et al. 2023; Daversa et al. 2022, 2025). For example, species declines have been illustrated in the southern corroboree frog ( *Pseudophryne corroboree* ) and northern corroboree frog ( *Pseudophryne pengilleyi* ) in the presence of the infected reservoir species *Crinia signifera* (Hunter et al. 2009; Hunter 2010; Scheele, Hunter, et al. 2017), which maintained Bd in the environment. In addition, the invasive North American Bullfrog (*Lithobates* [*Rana*] *catesbeianus*), also a reservoir species and a habitat generalist, has spread Bd over large areas causing declines in multiple other species (Garner et al. 2006; Yap et al. 2018). While these studies provide essential insights into individual susceptibility, context within a community framework remains less synthesised and comparatively underexplored relative to species‐level impacts, despite their importance for understanding transmission dynamics and informing Bd management (Blaustein et al. 2011; Brannelly, Clemann, et al. 2018; Daversa et al. 2022; Longo et al. 2023).

Mechanisms proposed to explain how Bd specifically influences amphibian communities fall into two non‐exclusive systems: (1) reservoir species maintain and transmit the fungus to susceptible species (Garner et al. 2006; Scheele, Hunter, et al. 2017); and (2) environmental persistence of free‐living Bd zoospores for up to 12 weeks sustains impacts on species (Chestnut et al. 2014). Studies focusing on explaining individual species susceptibility have emphasised genetic predispositions or life history traits (e.g., small geographic range and narrow environmental requirements) which might be risk factors for chytridiomycosis sensitivity (Lips et al. 2003; Bielby et al. 2008; Murray and Skerratt 2012; Savage et al. 2015; Burrowes et al. 2017). For example, hosts that are habitat generalists are more likely to act as reservoirs, while habitat specialists are more likely to be fatally susceptible (Lips et al. 2003; Bielby et al. 2008; Murray and Skerratt 2012). It is hypothesised that these relationships reflect evolutionary strategies of multi‐host pathogens towards maximising fitness by adapting to the most abundant host (Han et al. 2015). Building on this, theory suggests a combination of both direct effects (elicited by the infection of host species) and indirect effects (mediated through competition) can create novel interactions that either facilitate co‐existence or exacerbate declines (Bowers and Turner 1997; Holt et al. 2003; Parris and Cornelius 2004; Bosch and Rincón 2008; Dunn et al. 2012; Sapsford et al. 2015). These competitive interactions, together with favourable environmental conditions that enable persistence of Bd zoospores on hosts and in the environment (Murray and Skerratt 2012; Murray et al. 2013), can have dynamic repercussions for both disease persistence and community structure.

Here, we aim to advance understanding of Bd dynamics within wild amphibian communities by analysing long‐term data from amphibian assemblages in cool temperate Tasmania, Australia. Specifically, we test how Bd influences amphibian community structure through the mechanisms of differential susceptibility and potential competition (via shared microhabitat preferences) between species. Our study system comprises four co‐occurring frog species: the arboreal brown treefrog ( *Litoria ewingii* ) and Tasmanian treefrog ( *Litoria burrowsae* ), and the ground‐dwelling common froglet ( *Crinia signifera* ) and Tasmanian froglet ( *Crinia tasmaniensis* ). Both *L. ewingii* and *L. burrowsae* are arboreal species and *C. tasmaniensis* and *C. signifera* also have comparable life history traits; because they overlap in habitat preferences and share similar behaviour characteristics (Littlejohn 2003), it is hypothesised in our study that adults of these species may have potential for interspecific competition. Two of these species, *L. burrowsae* and *C. tasmaniensis* , are endemic and susceptible to Bd induced mortality in laboratory trials, whereas *L. ewingii* and *C. signifera* are widespread species and reservoirs of the pathogen (Littlejohn 2003; Lauck et al. 2005; Philips et al. 2010; Voyles et al. 2014; Scheele, Hunter, et al. 2017). We therefore expect the endemic susceptible species to decline in Bd‐positive sites, with further suppression where reservoir species are present and share the same microhabitat. By drawing on long‐term community level data, we offer context for understanding multi‐host pathogen dynamics in amphibian communities.

## 2 Methods

### 2.1 Study Region and Call Surveys

**FIGURE 1:**
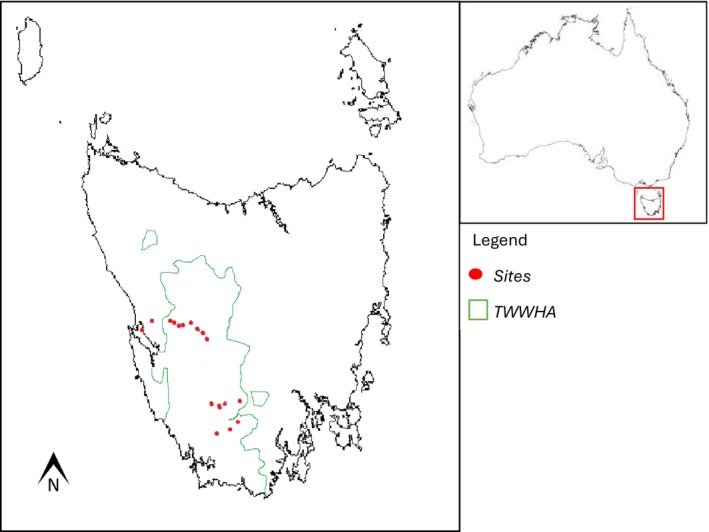
Amphibian survey sites were situated in Tasmania, Australia, across two regions: Lyell Highway region (west) and Strathgordon region (south), as denoted by the red dots. The Lyell Highway consists of 10 sites (L1–L10), and Strathgordon consists of 7 sites (S1–S7). The majority of sites are within the Tasmanian Wilderness World Heritage Area (TWWHA) which is outlined in green.

Study sites were situated in one of Australia's largest conservation areas, the Tasmanian Wilderness World Heritage Area (TWWHA) and were part of the Tasmanian *Department of Natural Resources and Environment* (NRE Tas) long term amphibian monitoring programme (Figure 1). Sites were originally selected on the basis of accessibility and records of previous *L. burrowsae* presence. This study included 17 pond sites across two regions, with 10 sites along the Lyell Highway (west TWWHA; L1–L10) and seven from Strathgordon (south TWWHA; S1–S7). The Lyell Highway sites were sampled from 2011 to 2016, and Strathgordon sites were sampled from 2011 to 2015. All sampling data were collected annually within the breeding season, which was the optimal time for detection, using call surveys, and Bd status was determined via the capture and swabbing of amphibians for Bd. Bd surveys were not conducted in all sites in all years, and therefore some years the status of a site was unknown (Appendix S1).

Environmental variables of pH and water temperature (°C) were measured at most sites in most years at least once and are used descriptively in this study. When recorded, water temperature (°C) and pH were sampled three times systematically at each pond site within 24 h of amphibian capture and sampling. Measurements were taken just below the water surface within each pond and averaged using a Handheld Digital Portable dual range pH meter and thermometer which were sterilised between uses.

In‐person call surveys or automated song recording units were used to assess community composition (methods described by Sinn and Philips 2014). In‐person call surveys were undertaken in 2011 and 2012, with observers listening for 5 min at least 30 min after sunset. From 2013 onwards, automated acoustic recording units (Song Meter SM2) sampled exactly 120 min after sunset for 5 min, and followed the same in‐person methodology, manually scoring species presence or absence for each call survey (Sinn and Philips 2014). There were at least three in‐person call surveys per site in 2011 and 2012. To ensure a greater than 95% probability of detecting a species if present at least five recordings per site per year are needed (Sinn and Philips 2014), and at most sites from 2013 this was accomplished with an average of six call surveys per site per year (Appendix S1). For analysis, we recorded whether a species was present or absent at a site within a year as occurrence. For site‐level descriptive results, we calculated the proportion of call surveys in which a species was present across all years.

### 2.2 Amphibian Skin Swabbing

Swabbing is a recommended sampling procedure for the detection of Bd in the field (Hyatt et al. 2007). To test for the presence of Bd at pond sites, tadpole and post‐metamorphic frog capture followed by swabbing was used. Call surveys were either completed before captures (2011–2012) or were performed on nights other than frog capture and swabbing (2013 onwards) to ensure no impact on acoustic surveys.

For the sampling of tadpoles, 20 or more *Litoria* spp. tadpoles per survey were randomly captured using small dipnets, and mouthparts were swabbed for Bd with a sterile swab (Pauza et al. 2010). The capture and swabbing of tadpoles were used in earlier surveys alongside frog swabbing (up to 2013), with adult frogs preferred in later years to detect Bd (2014 onwards).

Swab sampling of amphibians was conducted using standard Bd sampling techniques as described by Hyatt et al. (2007) and Sinn and Philips (2014). Physical searches for frogs were conducted 2 h after sunset (when frog activity is highest and thus locatable) for a period of 1 h, until all individuals were deemed to be captured or until 100 individuals were captured where density was high. Frogs were captured around the water's edge and in surrounding vegetation close to the pond sites. Each frog was captured using clean vinyl gloves and held individually in a sterile zip‐lock bag with sufficient air and water until processing. Briefly, after frog collections were completed, each frog was swabbed for the presence of Bd. To prevent cross contamination, new gloves were worn for sampling each individual. The sterile swab was brushed across the ventral side of the torso, inside of each of the front and back legs, and the pads of the hind and front feet five times per frog (Hyatt et al. 2007). Once the survey was complete, frogs were released at the site of capture, and biosecurity procedures were followed to minimise the risk of Bd spread (Sinn and Philips 2014).

Each swab sample was placed on ice in the field and then refrigerated before laboratory analysis. The refrigeration of dry swabs does not compromise Bd DNA detectability, even over extended storage periods (Hyatt et al. 2007), with samples in this study analysed within a month of collection. Samples were analysed at the NRE Tas Animal Health Laboratories in Mount Pleasant, Launceston, using quantitative real‐time TaqMan Polymerase Chain Reaction (PCR) assays, following the methodology of Hyatt et al. (2007). Samples were considered positive if any Bd DNA was present in the swab sample either pooled or individually tested. For the year 2016, Bd zoospores from swabs were quantified individually for all frogs which returned positive to determine the average Bd load.

### 2.3 Analysis

Prior to analysis, we evaluated distributions among Bd status, species occurrence, and environmental predictors to guide model specification and avoid over‐parameterisation. This assessment focused on identifying variables with limited independent variation using contingency tables, and highly associated predictors were not included together in the same models to ensure stable estimation and model convergence. All analysis were completed in R v4.1.0 (R Core Team 2021).

To evaluate whether our sampling effort in each year would have been sufficient to detect Bd across prevalence levels, where the number of individuals sampled was known, we calculated the minimum detectable prevalence (MDP) required to achieve 95% confidence of Bd calculated as: MDP = 1–0.05^(1/*n*)^, where *n* is the number of amphibians sampled in a given site–year. This quantifies the prevalence threshold above which Bd would be expected to be detected given the sampling effort in a given site and year.

We evaluated community and pathogen factors with the potential to influence the occurrence of each amphibian species across sites using a Bayesian Markov chain Monte Carlo (MCMC) mixed‐effects model within the package MCMCglmm (Hadfield 2010). For all models, the response variable was the species occurrence, defined as the presence or absence (0/1) of each amphibian at a given site in a given year. We included either Bd presence/absence based on swab samples for each site and year, or ongoing Bd presence/absence at the site (once detected, the site remained positive) as a fixed effect in the model. Because species with overlapping habitat use and life‐history traits may influence each other's occurrence, we included the species of the same genus within each species model as a fixed effect (Littlejohn 2003; Appendix S2). We also included an interaction term between Bd and the reservoir *L. ewingii* in models of *L. burrowsae* occurrence and between Bd and the reservoir *C. signifera* in models of *C. tasmaniensis* . Region (Lyell Highway or Strathgordon) was also included as a fixed effect. Random effects of Site (nested within region) and Year were included to account for non‐independence of observations within sites and across years.

We fitted two related models to address different ecological questions of Bd within amphibian communities. The primary model is presented in the main text, with an alternative model provided in Appendix S3. Our primary model tested the current association between Bd detection and species occurrence, evaluating whether Bd presence (0/1) based on swab results conducted that year and site influences amphibian occurrence. This model was restricted to sites and years in which Bd sampling was conducted. The second model assumed that sites remained Bd‐positive following first detection and was used as a sensitivity check to assess the robustness of the main model results to assumptions about Bd persistence.

Priors for random effects (Site, Year) were parameter‐expanded with weakly informative inverse‐Wishart distributions (*V* = 1, ν = 0.002), and residual variance was fixed to one, as required for categorical response models. This choice minimises prior influence while aiding mixing and convergence of variance parameters, a common issue when estimating small random effects in hierarchical models (Hadfield 2010). The model was run for ~13,000 iterations, with the first 3000 discarded as burn‐in, and every 10th retained, yielding ~1000 posterior distribution samples. Posterior means, 95% credible intervals, and effective sample sizes were extracted for inference. The proportion of variance explained by Site and Year was calculated as the variance of the random effect divided by the total variance (sum of all random effects and residual variance). Model convergence (including acceptance ratio and traceplots) was checked.

## 3 Results

Across ponds sites, average water temperature during winter to early spring (June to September) was 8°C (SD ±1.8°C), increasing to 15°C (SD ±2.4°C) during spring to early summer (October to January). In the Lyell Highway region, average water temperature during winter to early spring was 8°C (SD ±1.6°C), increasing to 15°C (SD ±3.4°C) in spring to early summer. In comparison, spring to early summer water temperatures for Strathgordon was 17°C (SD ±2.0°C). Mean pH across all sites was 5.0 (SD ±1.0), with Lyell highway sites averaging 4.8 (SD ±0.9), and Strathgordon sites averaging 5.6 (SD ±1.1). Summary tables of site pH and monthly water temperature across regions are provided in Appendix S4. While variable, all swab samples of Bd positive amphibians from 2016 had zoospore loads of less than 10,000 (Appendix S5).

Sampling effort varied substantially among years and sites. In some surveys, particularly after 2014, five or fewer post‐metamorphic frogs were swabbed, limiting detection to high Bd prevalences (> 40%–60%), whereas tadpole sampling in earlier years often allowed detection of lower prevalences due to larger sample sizes (Appendix S6). Low sample sizes should therefore be interpreted as inconclusive rather than evidence of true Bd absence.

**FIGURE 2:**
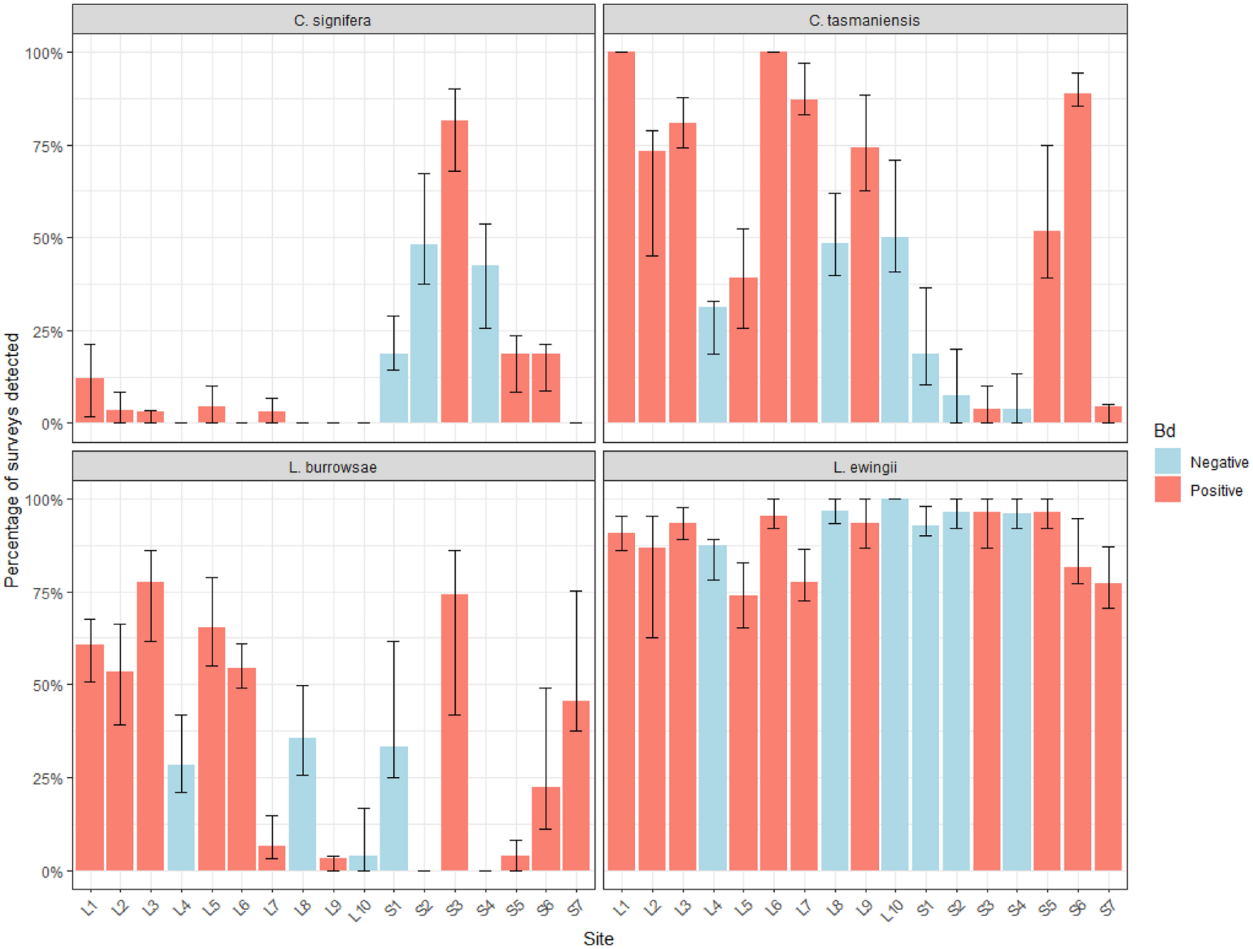
The percentage of call surveys each Tasmanian species was detected at each site from 2011 to 2016. The percentage was derived from the proportion of surveys the species was detected in for that site. The *x*‐axis indicates region and site, with Lyell Highway sites L1–L10 and Strathgordon sites S1–S7. The standard error bars show the variability in detection for each species call surveys over years for that site. In this figure, a site was classed as Bd positive if any amphibian swab was qPCR positive at least once across years, or Bd negative if it always returned negative from 2011 to 2016.

At the species level, *L. ewingii* and *C. tasmaniensis* were recorded across all sites, while *L. burrowsae* occurred at most sites (88%) and *C. signifera* was detected less frequently (65%). Within sites, *L. ewingii* was consistently recorded on 74% to 100% of survey occasions, and *L. burrowsae* was detected up to 77% of occasions at sites where it was present (Figure 2). In contrast, *C. tasmaniensis* was detected at least once at every site but showed greater temporal variability, particularly in the Strathgordon region (Figure 2). *Crinia signifera* was largely absent from surveys in the Lyell Highway region but was recorded across Strathgordon sites (Figure 2).

Bd was recorded at least once at 11 of 17 sites (65%) across the 5‐year period (Figure 2). Within the Lyell Highway region, Bd was recorded at 7 of 10 sites (70%), and in the Strathgordon region at 4 of 7 (57%). Sixty‐five percent of sites where *L. ewingii* and *C. tasmaniensis* were recorded were Bd positive (11 of 17 sites), and 73% of sites where *L. burrowsae* (11 of 15 sites) and *C. signifera* (8 of 11 sites) were recorded were also Bd positive.

**FIGURE 3:**
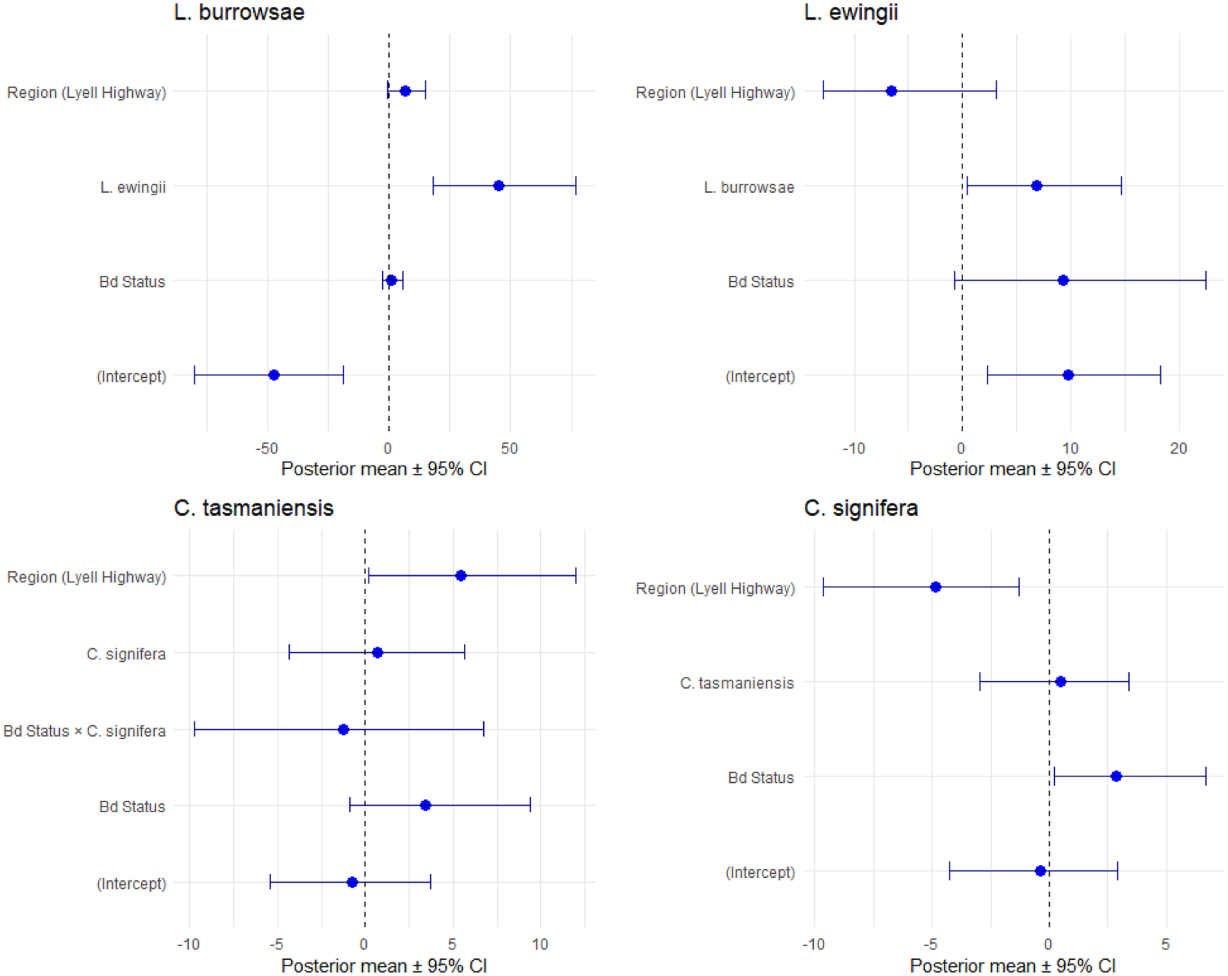
Models predicting the occurrence of the four amphibian species. Posterior mean estimates with 95% credible intervals for fixed effects from the four amphibian Bayesian MCMC models. Note that scales differ among models. *C. tasmaniensis* model includes the interaction term. The interaction term between *L. ewingii* and Bd status was removed in the *L. burrowsae* model due to non‐convergence. Each species model also includes the occurrence from its corresponding same‐genus species as a covariate (e.g., *L. ewingii* occurrence for the *L. burrowsae* MCMC model).

The primary and secondary model show similar findings for each species (Appendices S3 and S7). For the primary model, we initially considered an interaction between Bd status and the reservoir species *L. ewingii* for the *L. burrowsae* model; this term was not estimable due to a strong association between Bd occurrence and the presence of *L. ewingii* in the dataset and low model convergence. Consequently, the interaction term was excluded from the model. We found a trend for the occurrence of *L. burrowsae* to be positively associated with the presence of the sympatric species ( *L. ewingii* ) (posterior mean = 45.10, 95% CI: 18.128–77.024, pMCMC = < 0.001; Figure 3). Random effects suggested that Site accounted for most of the variation in *L. burrowsae* occurrence (posterior mean variance = 32.33, 95% CI: 1.293–81.81), corresponding to 92% of the total variance (95% credible interval: 65%–99%). While Year explained little (posterior mean = 1.073, 95% CI: < 0.001–5.2), corresponding to 3% of the total variance (95% credible interval: 0%–26%).

We found a weak positive association for the occurrence of *L. ewingii* to be positively associated with the presence of *L. burrowsae* at sites across years (posterior mean = 6.8, 95% CI: 0.431–14.597, pMCMC = 0.072). All other fixed effects overlapped zero (Figure 3). Random effects suggested that Site accounted for 10% (95% credible interval: 0%–79%) of variation in *L. ewingii* occurrence (posterior mean variance = 1.484, 95% CI: < 0.001–8.38). While Year contributed more (posterior mean variance = 34.82, 95% CI: < 0.001–138), corresponding to 66% of the total variance (95% credible interval: 0%–99%).

There was a significant positive effect of region on occurrence of *C. tasmaniensis* , with individuals more likely to occur at Lyell Highway sites (posterior mean = 5.428, 95% CI: 0.162–12.029, pMCMC = 0.026). Random effects suggested that Site accounted for a notable proportion of the variation in *C. tasmaniensis* occurrence (posterior mean variance = 16.58, 95% CI: 0.005–52.27), corresponding to 68% of the total variance (95% credible interval: 1%–98%). Year accounted for less variation (posterior mean variance = 2.897, 95% CI: < 0.001–13.01), with 12% of the total variance explained (95% credible interval: 0%–67%).

Finally, *C. signifera* was negatively associated with region, with individuals less likely to occur in Lyell Highway sites compared to Strathgordon (posterior mean = −4.834, 95% CI: −9.652–1.263, pMCMC = 0.018). We found the occurrence of *C. signifera* to be positively associated with the presence of Bd (posterior mean = 2.879, 95% CI: 0.197–6.677, pMCMC = 0.016). Random effects suggested that Site accounted for a small portion of variation in occurrence (posterior mean variance = 8.867, 95% CI: < 0.001–39.25), corresponding to 18% of the total variance (95% credible interval: 0%–76%). Year explained similar variation (posterior mean variance = 1.106, 95% CI: < 0.001–5.553) at 20% (95% credible interval: 0%–88%).

## 4 Discussion

Few studies have explored chytridiomycosis and differential susceptibility in amphibian communities with more than two species (Parris and Beaudoin 2004; Longo et al. 2023; Daversa et al. 2022, 2025). To address this gap, we examined the effect of Bd on wild amphibian communities comprising up to four species that vary in susceptibility which could lead to competition and pathogen induced declines. Despite evidence that two species in this assemblage ( *L. burrowsae* and *C. tasmaniensis* ) are susceptible to chytridiomycosis induced mortality (Obendorf and Dalton 2006; Voyles et al. 2014), we found no detectable effect of Bd or interspecific interactions on any species, apart from a weak positive association between *L. burrowsae* and *L. ewingii* occurrence. These findings indicate resilience of these seemingly susceptible amphibians to Bd under natural conditions (Scheele, Skerratt, et al. 2017). The lack of detectable pathogen or competition effects was unexpected, given laboratory trials demonstrating high Bd‐induced mortality in *L. burrowsae* (Voyles et al. 2014). However, this aligns with observations by Cashins et al. (2015), who found that sites with high *L. burrowsae* abundance were more likely to be Bd negative, although the species was still present at Bd positive sites. *Crinia signifera* was rarely detected in the Lyell Highway region; however, the positive association with Bd in the primary model suggests that the species may not be negatively affected by the pathogen, consistent with its role as a widespread reservoir host across eastern Australia (Hunter et al. 2009; Scheele, Hunter, et al. 2017). While *C. tasmaniensis* was more common in the absence of *C. signifera* , its occurrence did not appear to be influenced by *C. signifera* , or the interaction between Bd and the reservoir. The broad distribution of *L. ewingii* across all sites was unsurprising, reflecting its status as a generalist species throughout southeastern Australia (Lauck et al. 2005; Obendorf and Dalton 2006). Despite our models detecting limited associations, this study provides a rare insight into temperate amphibian community dynamics and highlights potential reasons why these communities might remain stable despite the presence of Bd.

Our results imply amphibian susceptibility to chytridiomycosis (often as determined in laboratory trials) represents only one component of risk to disease induced decline (Sapsford et al. 2015; Savage et al. 2015; Nowakowski et al. 2016). Laboratory experiments provide an important basis for understanding the pathophysiology of Bd in amphibian species, especially for species of conservation risk, such as those with restricted distributions or already threatened by habitat loss or climate change (Bielby et al. 2008; Carver et al. 2010; Voyles et al. 2014). While valuable, a key limitation of susceptibility trials is their extrapolation to disease development and outcomes in natural field settings (Kriger and Hero 2006). For example, laboratory conditions could induce stress in some species which interact with chytridiomycosis and ultimately compromise survival, whereas under natural settings the same Bd zoospore load may be sub‐lethal (Vredenburg et al. 2010; Rollins‐Smith 2016; Woodhams et al. 2011). Similarly, inoculum size of Bd zoospores tested in laboratory trials is often much higher (> 10,000 zoospores Vredenburg et al. 2010) relative to what amphibians might encounter in the wild (Kriger and Hero 2006; Savage et al. 2015). These differences are particularly well illustrated by Shaw et al. (2010) and Ohmer et al. (2013) on *L. ewingii* . Both studies reported *L. ewingii* as highly susceptible to chytridiomycosis under laboratory conditions when exposed to high zoospore inocula (~250,000) from non‐local Bd strains. Notably, Ohmer et al. (2013) also documented that field‐collected *L. ewingii* were Bd infected but asymptomatic, an observation supported elsewhere (Obendorf and Dalton 2006; Philips et al. 2010).

Development of chytridiomycosis, mass mortalities in the field, and rapid declines of amphibian populations are often associated with individuals whose Bd loads exceed a threshold of approximately 10,000 zoospore equivalents on their skin (Vredenburg et al. 2010; Voyles et al. 2012). Zoospore equivalents under this threshold are typically considered sub‐lethal infections and consequently not causing mortality. In our 2016 data, zoospore loads varied among Bd positive individuals within the same populations, but all were indicative of sub‐lethal infections. It remains unclear whether infection loads were persistently low across survey years or whether individuals with lethal loads were simply not detected. Nonetheless, zoospore loads may, alongside several other factors, help explain why susceptible species in this system did not appear to be strongly influenced by disease covariates within the community. Thus, low infection intensities across individuals could also reflect low environmental quantities of Bd within ponds (Chestnut et al. 2014; Chew et al. 2024), with estimates of Bd prevalence in this system likely to be at ~16% (as estimated in Pauza et al. 2010). Taken together, these patterns suggest that amphibian communities in this study might commonly experience sub‐lethal infection loads and low prevalence at pond sites.

Environmental mechanisms may play a greater role in shaping Bd dynamics in Tasmanian amphibian communities than previously anticipated. Savage et al. (2015) questioned the universal zoospore threshold, suggesting that it might only apply to certain species and that environmental conditions, irrespective of zoospore load, have a greater relative influence on the magnitude of Bd infection. Within laboratory settings, Bd grows between 4°C and 25°C, with optimum growth between 17°C and 25°C, highlighting temperature as an important driver of Bd ecology (Berger et al. 2004; Piotrowski et al. 2004). Seasonal infection patterns have been widely documented, with many studies reporting the greatest impacts during cooler months when temperatures fall within the optimal range for pathogen growth and survival (Berger et al. 2004; Piotrowski et al. 2004; Blaustein et al. 2012; Murray et al. 2013; Phillott et al. 2013; Brannelly et al. 2019; Wilber et al. 2022). In contrast, in temperate regions Bd growth may be optimal during warmer months, coinciding with the end of the amphibian breeding season. In the present study region, summer ambient temperatures (Lyell Highway: 5°C–19.4°C, 1900–2016; Strathgordon: 8.3°C–19.8°C, 1969–2015; Australian Bureau of Meteorology) and average pond temperature in late spring to early summer (15°C–17°C) align with Bd optimum growth, whereas winter air temperatures (Lyell Highway: 0°C–8.2°C, 1989–2016; Strathgordon: 3°C–10.1°C, 1969–2015; Australian Bureau of Meteorology) and average pond daytime temperatures (8°C) are less favourable for pathogen development (Piotrowski et al. 2004). Consequently, Bd might be suppressed during the early breeding season, with associated chytridiomycosis mortality instead occurring later in summer, outside of the period typically captured by the call surveys.

Additionally, the proposed environmental tolerance framework (Nowakowski et al. 2016) suggests that hosts adapted to conditions suboptimal for Bd are more likely to persist and avoid severe pathology. Accordingly, this is consistent with the relationship between temperature and breeding season described above and emphasises the role of the thermal environment in shaping the dynamics of disease within amphibian communities (Pounds et al. 2006; Hawley and Altizer 2011; Savage et al. 2015; Nowakowski et al. 2016; Sonn et al. 2020; Waddle et al. 2024). Because temperature influences both host body temperature and environmental suitability for Bd, the cool body temperatures of Tasmanian amphibians might physiologically constrain Bd for much of the year, contributing to low infection rates and the absence of obvious declines. Other factors are also likely important. For example, pond pH might limit Bd growth, as large parts of the TWWHA are naturally acidic (pH 5), outside Bd's optimal growth range (pH 6–7), thereby reducing zoospore survival, zoospore load on the host, and infection prevalence (Piotrowski et al. 2004; Pauza et al. 2010; Blaustein et al. 2012; Chestnut et al. 2014; Simpkins et al. 2017). Additionally, host behaviour might also play a role, with arboreal species such as *L. burrowsae* and *L. ewingii* spending much of their time out of the water, reducing exposure to free‐living Bd relative to aquatic species (Burrowes et al. 2017; Brannelly et al. 2021). Collectively, the interplay between low temperature, acidic ponds, and behavioural differences likely constrain Bd growth, reducing zoospore loads, and resulting in sub‐lethal effects on susceptible species without driving obvious population declines.

Although Bd, implicated by reservoir hosts, has been associated with the extinction or decline of nearly one‐fifth of Australian frog species, temperate Australian frogs do not always exhibit abrupt Bd‐driven declines (Scheele, Skerratt, et al. 2017). Many temperate species persist through mechanisms that reduce exposure or mitigate infection. For example, species that survive in cold winter climates might terrestrially overwinter or use arboreal microhabitats, reducing contact with aquatic zoospores and slowing transmission relative to stream or wetland taxa (Burrowes et al. 2017; Scheele, Skerratt, et al. 2017). Other diverse mechanisms include behavioural avoidance of diseased individuals (McMahon et al. 2014), potential self‐medication (Shaw et al. 2010), shifts in microbial community structure on amphibian skin (Woodhams et al. 2006; Bletz et al. 2017), and innate or adaptive immune responses (McMahon et al. 2014; Knapp et al. 2016). The thermal mismatch framework and Bd suitability models further suggest that hosts adapted to conditions outside Bd's performance optimum experience less severe pathology (Murray et al. 2013; Nowakowski et al. 2016; Cohen et al. 2017; Carvalho et al. 2024; Thumsová et al. 2025). For example, physical characteristics of waterbodies such as elevated salinity have been shown to reduce Bd prevalence in *L. raniformis* in south‐eastern Australia (Heard et al. 2014, 2015). Together, these mechanisms help explain why many susceptible, temperate species persist with low, often sub‐lethal infection loads, display stable or gradual population changes, and show spatially variable Bd prevalence. However, caution is warranted when interpreting results as declines can be slow, seasonally masked, or emerge as climates shift towards Bd‐favourable conditions (Voyles et al. 2012; Scheele, Skerratt, et al. 2017; Scheele et al. 2019; Wilber et al. 2022). In addition, changes not directly measured here, such as altered population age structure or demographic shifts, might already be occurring (Scheele et al. 2015, 2016). For instance, high adult mortality from chytridiomycosis could be partially offset by high recruitment of juveniles (Phillott et al. 2013), allowing populations to persist despite seasonal die‐offs, at least in the short term.

This study provides rare insight into whether Bd influences a multi‐host amphibian community within a temperate region. We suggest that the discrepancies between laboratory trials and natural outcomes could reflect environmental contexts; particularly the low temperatures, acidic tannin‐rich waters, and terrestrial behaviours that together constrain Bd growth, reduce zoospore persistence, and buffer vulnerable species. In this sense, the TWWHA could function as an infected refuge, where sub‐lethal infections persist but declines are not yet apparent (Puschendorf et al. 2009; Skerratt et al. 2016; Heard et al. 2015; Cohen et al. 2017; Kärvemo et al. 2018). However, limited surveys conducted before the first detection of Bd in Tasmania (2004) mean that the pathogen could have arrived years earlier and was already enzootic when surveys commenced (Phillips et al. 2012; Cashins et al. 2015). Further complexities arise when evaluating abundance from call surveys since species may still be present at a site even if the population has declined. Therefore, detecting Bd effects on amphibian populations might be difficult (Puschendorf et al. 2009), a challenge common to many amphibian populations across Australia and worldwide (Lips et al. 2006; Phillips et al. 2012). This challenge is compounded by low expected Bd prevalence (~16%; Pauza et al. 2010) and limited detection power in years or sites with small sample sizes. Therefore, future studies should prioritise increased sampling and detection effort to reliably estimate infection status and disentangle low prevalence from true pathogen absence. Future research avenues should focus on zoospore load and environmental variables such as pH and water temperature and their added influence on Bd within a community structure. Our findings highlight that future warming could shift Bd dynamics towards the pathogen's thermal optimum, increasing outbreak risk and weakening the buffering effect of current environmental conditions (Pounds et al. 2006; Bosch et al. 2007; McMenamin et al. 2008; Blaustein et al. 2012; Rollins‐Smith 2016; Cohen et al. 2017, 2020). Thus, while our results suggest relative resilience of Tasmanian amphibians, this stability could be transient. Long‐term monitoring, combined with field experiments and community context studies of pathogen persistence, will be critical for anticipating climate‐driven shifts, informing conservation strategies, and safeguarding endemic amphibian species such as *L. burrowsae* .

## Author Contributions

**Elise Ringwaldt:** conceptualization (equal), data curation (equal), formal analysis (lead), project administration (equal), writing – original draft (lead), writing – review and editing (equal). **Shannon Troy:** conceptualization (equal), data curation (equal), formal analysis (equal), project administration (equal), supervision (equal), writing – review and editing (equal). **Annie Philips:** conceptualization (equal), data curation (equal), funding acquisition (equal), project administration (equal), writing – review and editing (equal). **Scott Carver:** conceptualization (equal), data curation (equal), formal analysis (equal), funding acquisition (equal), project administration (equal), supervision (equal), writing – review and editing (equal).

## Funding

Funding was provided by the Department of Natural Resources and Environment Tasmania (NRE Tas, formerly DPIPWE) and the Tasmanian Government Scholarship in Wildlife Conservation (NRE Tas and University of Tasmania).

## Conflicts of Interest

The authors declare no conflicts of interest.

## Supporting information


**Data S1:** ece373201‐sup‐0001‐DataS1.docx.

## Data Availability

The data to support the findings of this study are available in Supporting Information. Certain restrictions may apply to the availability of additional data. For specifics, please consult the corresponding author.
