# Supplementary material for: Differential Susceptibility May Not Drive Chytridiomycosis Related Declines in Multi‐Host Amphibian Communities
Source: Ecol Evol. 2026 Mar 8;16(3):e73201. doi: 10.1002/ece3.73201 (PMC12967501; doi:10.1002/ece3.73201)
Supplement: Supplementary file 1 — Data S1: ece373201‐sup‐0001‐DataS1.docx. [file ECE3-16-e73201-s001.docx]

**Differential Susceptibility May Not Drive Chytridiomycosis Related Declines in Multi‐Host Amphibian Communities**

**Elise Ringwaldt^1*^, Shannon Troy^2^, Annie Philips^2^, Scott Carver^1,3^.**

Affiliations:

1. University of Tasmania, School of Natural Sciences, Hobart, AUS

2. The Department of Natural Resources and Environment, Tasmanian Government, Hobart AUS

3. University of Georgia, Odem School of Ecology, Athens, GA, USA

*Corresponding Author: elise.ringwaldt@utas.edu.au.

Appendicies.

**Appendix 1.** **Each site and year and the corresponding number of acoustic surveys and Bd swab surveys for the Lyell Highway (L1-L10) and Strathgordon (S1-S7) regions.**

Acoustic: the number of amphibian call surveys completed each year per site. swabBd: Whether amphibians were swab sampled for Bd that year and the corresponding Bd result of either positive (1) or negative (0), NA indicates no sampling was done for that site and year. Bdpersist: the corresponding site status if once a site records a positive it remains positive. Some sites were not assessed equally or every year due to resources, field or recording-unit difficulties (NA).

| **Site** | **Survey** | **2011** | **2012** | **2013** | **2014** | **2015** | **2016** |
| --- | --- | --- | --- | --- | --- | --- | --- |
| **L1** | **Acoustic** | 4 | 4 | 10 | 5 | 5 | 5 |
|  | **swabBd** | 0 | 0 | 1 | NA | 0 | 0 |
|  | **Bdpersist** | 0 | 0 | 1 | 1 | 1 | 1 |
| **L2** | **Acoustic** | 3 | 4 | 10 | 5 | 3 | 5 |
|  | **swabBd** | 0 | 1 | NA | NA | 0 | 1 |
|  | **Bdpersist** | 0 | 1 | 1 | 1 | 1 | 1 |
| **L3** | **Acoustic** | 3 | 3 | 10 | 5 | 5 | 5 |
|  | **swabBd** | 0 | 0 | NA | 0 | 1 | 1 |
|  | **Bdpersist** | 0 | 0 | 0 | 0 | 1 | 1 |
| **L4** | **Acoustic** | 4 | 3 | 10 | 5 | 5 | 5 |
|  | **swabBd** | NA | 0 | NA | 0 | NA | 0 |
|  | **Bdpersist** | NA | 0 | 0 | 0 | 0 | 0 |
| **L5** | **Acoustic** | 4 | 4 | NA | 5 | 5 | 5 |
|  | **swabBd** | 0 | 1 | 1 | NA | 1 | 0 |
|  | **Bdpersist** | 0 | 1 | 1 | 1 | 1 | 1 |
| **L6** | **Acoustic** | 4 | 4 | NA | 4 | 5 | 5 |
|  | **swabBd** | 0 | 0 | 1 | NA | 0 | 0 |
|  | **Bdpersist** | 0 | 0 | 1 | 1 | 1 | 1 |
| **L7** | **Acoustic** | 3 | 3 | 10 | 5 | 5 | 5 |
|  | **swabBd** | 0 | NA | NA | 1 | NA | 1 |
|  | **Bdpersist** | 0 | NA | NA | 1 | 1 | 1 |
| **L8** | **Acoustic** | 4 | 3 | 9 | 5 | 5 | 5 |
|  | **swabBd** | 0 | 0 | NA | NA | 0 | 0 |
|  | **Bdpersist** | 0 | 0 | 0 | 0 | 0 | 0 |
| **L9** | **Acoustic** | 4 | 3 | 9 | 5 | 5 | 5 |
|  | **swabBd** | NA | 0 | NA | 1 | 1 | 1 |
|  | **Bdpersist** | 0 | 0 | NA | 1 | 1 | 1 |
| **L10** | **Acoustic** | 3 | 3 | 10 | NA | NA | 5 |
|  | **swabBd** | 0 | 0 | NA | 0 | NA | NA |
|  | **Bdpersist** | 0 | 0 | 0 | 0 | NA | NA |
| **S1** | **Acoustic** | 3 | 4 | 10 | 5 | 5 | NA |
|  | **swabBd** | 0 | NA | NA | NA | 0 | NA |
|  | **Bdpersist** | 0 | 0 | 0 | 0 | 0 | NA |
| **S2** | **Acoustic** | 3 | 4 | 10 | 5 | 5 | NA |
|  | **swabBd** | 0 | 0 | NA | 0 | 0 | NA |
|  | **Bdpersist** | 0 | 0 | 0 | 0 | 0 | NA |
| **S3** | **Acoustic** | 3 | 4 | 10 | 5 | 5 | NA |
|  | **swabBd** | 1 | 1 | 1 | NA | NA | NA |
|  | **Bdpersist** | 1 | 1 | 1 | 1 | 1 | 1 |
| **S4** | **Acoustic** | 3 | 4 | 9 | 5 | 5 | NA |
|  | **swabBd** | 0 | NA | NA | NA | 0 | NA |
|  | **Bdpersist** | 0 | 0 | 0 | 0 | 0 | NA |
| **S5** | **Acoustic** | 3 | 4 | 10 | 5 | 5 | NA |
|  | **swabBd** | NA | 0 | NA | 0 | 1 | NA |
|  | **Bdpersist** | 0 | 0 | 0 | 0 | 1 | 1 |
| **S6** | **Acoustic** | 3 | 4 | 10 | 5 | 5 | NA |
|  | **swabBd** | NA | NA | NA | 1 | NA | NA |
|  | **Bdpersist** | NA | NA | NA | 1 | 1 | 1 |
| **S7** | **Acoustic** | NA | 3 | 10 | 4 | 5 | NA |
|  | **swabBd** | NA | 0 | NA | NA | 1 | NA |
|  | **Bdpersist** | 0 | 0 | NA | NA | 1 | 1 |

**Appendix 2.Tasmanian amphibian life history, ecology, and suceptibility.**

Our study system most frequently consists of four frog species: the arboreal brown treefrog (*Litoria ewingii*) and Tasmanian tree frog (*Litoria burrowsae*), the ground-dwelling common froglet (*Crinia signifera*) and Tasmanian froglet (*Crinia tasmaniensis*). Competitive interactions among these species are not well understood. Both *L. ewingii* and *L. burrowsae* are arboreal species and, because they overlap in habitat preferences and share similar behaviour characteristics (Littlejohn 2003), it is hypothesised in our study that adults of these species may compete. Similarly, *C. tasmaniensis* and *C. signifera* also have comparable life history traits, are ground-dwelling amphibians, sharing habitat in both larval and adult phases (Littlejohn 2003), and thus have potential for interspecific competition.

*Litoria ewingii* is arboreal, is a habitat generalist and breeding opportunistic to large periods of rainfall (Littlejohn 2003). Few factors restrict its choice of breeding sites making it a very successful species that is common and widespread across Tasmania and other parts of Australia (Lauck et al. 2005). *L. ewingii* is generally considered a reservoir species as multiple studies have found it to be widely tolerant to Bd exposure and commonly found in areas where Bd persists (Obendorf and Dalton 2006; Pauza et al. 2010; Philips et al. 2010). Some laboratory studies have contrasted with this, but it should be noted that these have been though exposure to novel strains and very high zoospore loads (Shaw et al. 2010; Ohmer et al. 2013).

*Litoria burrowsae* is a Tasmanian endemic arboreal species predominately found throughout the west and south-west of the state (Littlejohn 2003; Voyles et al. 2014; Skerratt et al. 2016). *L. burrowsae* mainly breeds in spring and summer in stationary or slowly flowing water, but are opportunistic breeders with large rainfall also (Littlejohn 2003). Laboratory studies have shown *L. burrowsae* to be highly susceptible to chytridiomycosis with 100% mortality (n=3) within 30 days of initially exposed (exposure to 1.5 × 10^6^ Bd zoospores, Voyles et al. 2014), supporting suggestions of this species to be at high risk of declining (Obendorf and Dalton 2006; Voyles et al. 2014; Cashins et al. 2015).

*Crinia signifera* are small ground dwelling frogs, which breed throughout most of the year, are widespread throughout Tasmania, and like *L. ewingii* are also habitat generalists making them a very successful group of amphibians across parts of Australia. Numerous study have also described *C. signifera* as a reservoir, obtaining high Bd loads in the field without decline (Hunter et al. 2009; Hunter 2010; Brannelly et al. 2017). Scheele, Hunter, et al. (2017) supported *C. signifera* as tolerant to Bd infection with a 12-week laboratory study.

*Crinia tasmaniensis* has a very similar appearance to *C. signifera* but has red hue or patches on the ventral surfaces of the thighs, flanks and abdomen. *C. tasmaniensis* is endemic to Tasmania, restricted to moister parts of the state (Littlejohn 2003). Studies by Obendorf (2005) have shown *C. tasmaniensis* to also be susceptible to chytridiomycosis with infected juvenile amphibians dying 4-6 weeks after metamorphose (but see Philips et al. 2010 and Voyles et al. 2014, who have described the potential susceptibility as unknown).

Additional Reference:

Obendorf, D. 2005. *Application of Field and Diagnostic Methods for Chytridiomycosis in Tasmanian Frogs*. Vol. 35. Central North Field Naturalists Inc.

**Appendix 3.** **In this model, once a site was detected as Bd positive, that site remained positive.**

The persistence of Bd in a susceptible population may happen (even at low levels) due to the presence of reservoir species, which maintain Bd in the environment, and the persistence of aquatic zoospores (Woodhams et al. 2011). Therefore, in our study, Bd could persist in a site even at low prevalence, particularly since we have two reservoir species within the amphibian community. This MCMCglmm model allowed us to include years preceding Bd positive detection at a site even when swabbing was not performed but call surveys were.

All Posterior mean estimates with 95% credible intervals for fixed effects, and posterior mean estimates for random effects, from the four amphibian Bayesian MCMC models. Model convergence (traceplots) was checked. Note that scales differ among models. The *L. ewingii* and *C. signifera* models do not include the interaction effect. The interaction term between *L. ewingii* and Bd status was removed in the *L. burrowsae* model due to non-convergence. Each species model also includes the occurrence from its corresponding same-genus species as a covariate. Trace plots were checked for all models.

| MCMC |  |  |  |  |  |  |  |  |  |
| --- | --- | --- | --- | --- | --- | --- | --- | --- | --- |
| Iterations | 3001:12991 |  |  |  |  |  |  |  |  |
| Thinning interval | 10 |  |  |  |  |  |  |  |  |
| Sample size | 1000 |  |  |  |  |  |  |  |  |
| Residual | 1 |  |  |  |  |  |  |  |  |
|  | ***C. signifera*** | | |  |  | ***L. ewingii*** | | |  |
|  | mean | low-95% CI | upper-95% CI | |  | mean | low-95% CI | upper-95% CI | |
| Site Nested (RE) | 0.5552 | 0.000306 | 2.636 |  | **Site Nest (RE)** | 25.73 | 0.000308 | 90.14 |  |
| Year (RE) | 0.7744 | 0.000251 | 3.401 |  | **Year (RE)** | 1.332 | 0.000344 | 7.639 |  |
| site prop | 0.173195 | 0.000779 | 0.703989 |  | **site prop** | 0.611051 | 0.000752 | 0.988787 |  |
| year prop | 0.203324 | 0.000738 | 0.796184 |  | **year prop** | 0.018002 | <0.0001 | 0.164541 |  |
|  |  |  |  | pMCMC |  |  |  |  | pMCMC |
| (Intercept) | 0.7846 | -0.9576 | 2.3068 | 0.34 | **(Intercept)** | 21.1215 | 7.9241 | 40.2172 | <0.001* |
| Bd (persist) | 1.6561 | -0.153 | 3.9214 | 0.07 | **Bd (persist)** | -8.5601 | -18.8755 | 1.2282 | 0.116 |
| C. tasmaniensis | 0.073 | -1.6663 | 2.1957 | 0.962 | **Lb Occurrence** | 15.5799 | 0.8227 | 28.3862 | 0.046* |
| Region (Two) | -4.4065 | -6.664 | -2.4295 | <0.001* | **Region (Lyell Highway)** | -9.5529 | -20.5357 | 2.3055 | 0.104 |
|  |  |  |  |  |  |  |  |  |  |
|  | ***C. tasmaniensis*** | | |  |  | ***L. burrowsae*** | | |  |
|  | mean | low-95% CI | upper-95% CI | |  | mean | low-95% CI | upper-95% CI | |
| Site Nested (RE) | 7.152 | 0.000333 | 20.79 |  | **Site Nested (RE)** | 34.78 | 1.112 | 117.9 |  |
| Year (RE) | 0.3331 | 0.000174 | 1.649 |  | **Year (RE)** | 0.3841 | 0.000224 | 1.523 |  |
| site prop | 0.724168 | 0.00689 | 0.956386 |  | **site prop** | 0.905723 | 0.660118 | 0.992273 |  |
| year prop | 0.041711 | <0.0001 | 0.350129 |  | **year prop** | 0.018002 | <0.0001 | 0.164541 |  |
|  |  |  |  | pMCMC |  |  |  |  | pMCMC |
| (Intercept) | -0.1696 | -3.4344 | 2.9047 | 0.926 | **(Intercept)** | -35.185 | -75.994 | -4.897 | <0.001* |
| Bd (persist) | -1.3678 | -5.0618 | 2.3716 | 0.486 | **Bd (persist)** | 1.674 | -1.187 | 5.306 | 0.272 |
| Cs Occurrence | 0.0101 | -2.7855 | 2.888 | 1 | **Le Occurrence** | 33.929 | 4.539 | 74.333 | 0.004* |
| Region (Lyell Highway) | 5.5319 | 1.962 | 9.9131 | 0.004* | **Region (Lyell Highway)** | 4.514 | -1.263 | 13.366 | 0.104 |
| Bd * Cs interaction | 2.4697 | -3.1046 | 8.1496 | 0.394 | **NA – no interaction** |  |  |  |  |

**Appendix 4.** **Summary tables of site pH and monthly water temperature across regions.**

**Table 1:** Showing the average water temperature for each month across all sites and then separate for each region. The number of site surveys for which we had sampled water temperature, the average water temperature for each month and standard deviation of the mean. Total is across all regions, and then each region is also shown separately. Note that Strathgordon only had surveys/samples during late spring to early summer, with no winter surveys conducted.

| **TOTAL** |  |  |  |
| --- | --- | --- | --- |
| **Month** | **Number of surveys** | **Average Water Temp °C** | **StdDev of Water Temp °C** |
| January | 11 | 18.1 | 2.9 |
| February | 2 | 19.3 | 1.2 |
| April | 1 | 11.0 | NA |
| May | 2 | 9.0 | 2.5 |
| June | 2 | 6.7 | 4.0 |
| July | 10 | 6.5 | 0.8 |
| August | 1 | 7.5 | NA |
| September | 31 | 10.5 | 2.2 |
| October | 12 | 13.8 | 2.5 |
| November | 6 | 12.7 | 3.1 |
| December | 5 | 15.6 | 3.1 |
| Grand Total | 83 | 12.0 | 4.3 |
| **LYELL HIGHWAY** |  |  |  |
| **Month** | **Number of surveys** | **Average of Water Temp C** | **StdDev of Water Temp C** |
| January | 6 | 18.9 | 3.4 |
| February | 2 | 19.3 | 1.2 |
| April | 1 | 11.0 | NA |
| May | 2 | 9.0 | 2.5 |
| June | 2 | 6.7 | 4.0 |
| July | 10 | 6.5 | 0.8 |
| August | 1 | 7.5 | NA |
| September | 25 | 9.9 | 1.9 |
| October | 11 | 13.3 | 1.8 |
| November | 5 | 11.6 | 1.6 |
| December | 2 | 17.1 | 0.0 |
| Total | 67 | 11.2 | 4.2 |
| **STRATHGORDON** |  |  |  |
| **Month** | **Number of surveys** | **Average of Water Temp C** | **StdDev of Water Temp C** |
| January | 5 | 17.2 | 2.2 |
| September | 6 | 12.9 | 1.5 |
| October | 1 | 19.4 | NA |
| November | 1 | 18.2 | NA |
| December | 3 | 14.6 | 3.9 |
| Total | 16 | 15.3 | 3.1 |

**Table 2:** Showing the average pH of each site, each year that was collected with a Handheld Digital Portable dual range pH meter. pH was averaged if there was more than one reading within the year. NA if there were no readings during the year. The final column shows the overall pH average for the site across all years. For L2 in 2012 only one sample was collected that year, and due to no replication (and low replication across years and sites in some instances except 2016), pH is used as descriptive results.

| **Site** | **2011** | **2012** | **2013** | **2014** | **2015** | **2016** | **Site Average** |
| --- | --- | --- | --- | --- | --- | --- | --- |
| **L1** | 5.20 | 4.94 | 4.54 | NA | 4.67 | 4.57 | 4.78 |
| **L2** | 4.55 | 8.68 | NA | NA | NA | 4.34 | 5.86 |
| **L3** | 4.70 | 4.64 | NA | 4.55 | 4.47 | 4.63 | 4.60 |
| **L4** | 4.55 | 4.67 | NA | 4.43 | NA | 4.32 | 4.49 |
| **L5** | 4.61 | 4.89 | 4.72 | NA | NA | 4.37 | 4.65 |
| **L6** | 4.35 | 5.06 | 4.14 | NA | 4.02 | 4.38 | 4.39 |
| **L7** | 4.77 | 8.79 | NA | 4.65 | NA | 4.92 | 5.78 |
| **L8** | 4.75 | 4.85 | NA | NA | 4.40 | 4.83 | 4.71 |
| **L9** | 4.65 | 4.63 | NA | 4.54 | 4.46 | 4.49 | 4.55 |
| **L10** | 4.79 | 4.64 | NA | 4.60 | NA | 4.60 | 4.66 |
| **S1** | 4.68 | 5.84 | NA | NA | 5.70 | NA | 5.41 |
| **S2** | 4.73 | 4.72 | NA | 4.86 | 6.50 | NA | 5.20 |
| **S3** | 6.96 | 7.32 | NA | NA | NA | NA | 7.14 |
| **S4** | 5.39 | 8.53 | NA | NA | 4.70 | NA | 6.21 |
| **S5** | 4.70 | 5.73 | NA | 4.68 | 4.55 | NA | 4.91 |
| **S6** | 6.20 | NA | NA | 6.40 | NA | NA | 6.30 |
| **S7** | NA | NA | NA | NA | 4.46 | NA | 4.46 |

**Table 3:** More detailed systematic sampling year. Water temperature and pH results across pond survey sites in the Lyell Highway Region in 2016. Site ID indicates the sample site. Survey indicates when sampling was conducted: 1 (July) or 2 (September). Water temperature and pH during the day of sampling are shown. The results of the swab samples of amphibian skin taken within a 24 hour period for both sampling occasions, with 0 representing negative and 1 positive. NA indicates that the pond site was not surveyed for an occasion (Site: L5), or that no amphibians were captured and therefore there was no PCR swab test result (Site: L3, L8, L10).

|  | | |  |  | | |  |  | |  |
| --- | --- | --- | --- | --- | --- | --- | --- | --- | --- | --- |
| Site ID | Survey | Water temperature °C | | | pH | Amphibians sampled | | | Bd Result (Swabbing) | |
| L1 | 1 | 5.9 | | | 4.67 | 2 | | | 0 | |
|  | 2 | 9.9 | | | 4.47 | 2 | | | 0 | |
| L2 | 1 | 6.2 | | | 4.22 | 6 | | | 0 | |
|  | 2 | 8.3 | | | 4.45 | 14 | | | 1 | |
| L3 | 1 | 7.5 | | | 4.68 | 0 | | | NA | |
|  | 2 | 11.2 | | | 4.58 | 11 | | | 1 | |
| L4 | 1 | 6.5 | | | 4.27 | 1 | | | 0 | |
|  | 2 | 11.2 | | | 4.36 | 2 | | | 0 | |
| L5 | 1 | 4.9 | | | 4.33 | NA | | | NA | |
|  | 2 | 5.5 | | | 4.40 | 7 | | | 0 | |
| L6 | 1 | 6.2 | | | 4.29 | 3 | | | 0 | |
|  | 2 | 6.0 | | | 4.46 | 4 | | | 0 | |
| L7 | 1 | 6.1 | | | 4.55 | 19 | | | 1 | |
|  | 2 | 8.0 | | | 5.29 | 16 | | | 1 | |
| L8 | 1 | 6.9 | | | 4.82 | 0 | | | NA | |
|  | 2 | 8.6 | | | 4.83 | 5 | | | 0 | |
| L9 | 1 | 7.0 | | | 4.26 | 5 | | | 0 | |
|  | 2 | 10.6 | | | 4.72 | 5 | | | 1 | |
| L10 | 1 | 7.6 | | | 4.68 | 0 | | | NA | |
|  | 2 | 10.0 | | | 4.51 | 0 | | | NA | |

**Appendix 5.** The number of Bd zoospores each positive individual amphibian was carrying in the 2016 amphibian swab surveys. In total, nine amphibians tested positive for Bd over both sampling occasions. All Bd loads were regarded as ‘sub-lethal' with <10,000 zoospore count. We acknowledge the low sample size of individual zoospore testing which is not consistently available across all years in the dataset.

| **Zoospore count for Bd positive amphibians** | | |
| --- | --- | --- |
| Amphibian ID | Species | Zoospore Count |
| 1 | *C. tasmaniensis* | 197 |
| 2 | *C. tasmaniensis* | 90 |
| 3 | *C. tasmaniensis* | 153 |
| 4 | *L. ewingii* | 335 |
| 5 | *L. ewingii* | 619 |
| 6 | *L. ewingii* | 718 |
| 7 | *L. ewingii* | 1363 |
| 8 | *L. ewingii* | 2220 |
| 9 | *L. ewingii* | 7820 |

**Appendix 6.** Minimum detectable prevalence (MDP) of *Batrachochytrium dendrobatidis* at 95% confidence for different amphibian sample sizes in this study, calculated as: MDP = 1 − 0.05^(1/n). MDP represents the lowest true prevalence that would be detected with 95% probability given the number of individuals sampled in a site and year.

| **Number of amphibians** | **MDP 95%** |
| --- | --- |
| 2 | 0.7764 |
| 3 | 0.6316 |
| 4 | 0.5271 |
| 5 | 0.4507 |
| 6 | 0.3930 |
| 7 | 0.3482 |
| 8 | 0.3123 |
| 9 | 0.2831 |
| 10 | 0.2589 |
| 11 | 0.2384 |
| 12 | 0.2209 |
| 13 | 0.2058 |
| 20 | 0.1391 |
| 21 | 0.1329 |
| 23 | 0.1221 |
| 24 | 0.1173 |
| 25 | 0.1129 |
| 34 | 0.0843 |
| 40 | 0.0722 |
| 47 | 0.0618 |
| 81 | 0.0363 |

**Appendix 7**. All Posterior mean estimates with 95% credible intervals for fixed effects, and posterior mean estimates for random effects, from the four amphibian Bayesian MCMC models. Model convergence (traceplots) was checked. Note that scales differ among models. The *L. ewingii* and *C. signifera* models do not include the interaction effect. The interaction term between *L. ewingii* and Bd status was removed in the *L. burrowsae* model due to non-convergence. Each species model also includes the occurrence from its corresponding same-genus species as a covariate. Trace plots were checked for all models. For *C. signifera,* traceplots showed slow mixing due to sparse data and unevenness in the occurrence of this species across sites and region, however posterior summaries were stable across acceptance ratios (~0.45).

| MCMC |  |  |  |  |  |  |  |  |  |
| --- | --- | --- | --- | --- | --- | --- | --- | --- | --- |
| Iterations | 3001:12991 |  |  |  |  |  |  |  |  |
| Thinning interval | 10 |  |  |  |  |  |  |  |  |
| Sample size | 1000 |  |  |  |  |  |  |  |  |
| Residual | 1 |  |  |  |  |  |  |  |  |
|  | ***C. signifera*** | | |  |  | ***L. ewingii*** | | |  |
|  | mean | low-95% CI | upper-95% CI | |  | mean | low-95% CI | upper-95% CI | |
| Site Nested (RE) | 8.867 | 0.000478 | 39.25 |  | **Site Nest (RE)** | 1.484 | 0.000185 | 8.38 |  |
| Year (RE) | 1.106 | 0.000289 | 5.553 |  | **Year (RE)** | 34.82 | 0.000514 | 138 |  |
| site prop | 0.183463 | 0.000696 | 0.755001 |  | **site prop** | 0.102690 | 0.000022 | 0.788025 |  |
| year prop | 0.198128 | 0.000600 | 0.875536 |  | **year prop** | 0.661031 | 0.001737 | 0.993487 |  |
|  |  |  |  | pMCMC |  |  |  |  | pMCMC |
| (Intercept) | -0.3878 | -4.259 | 2.9014 | 0.818 | **(Intercept)** | 9.75 | 2.2844 | 18.2239 | 0.004* |
| Bd Status | 2.8787 | 0.1967 | 6.6769 | 0.016* | **Bd Status** | 9.3185 | -0.7109 | 22.4236 | 0.13 |
| C. tasmaniensis | 0.4957 | -2.9452 | 3.4114 | 0.742 | **Lb Occurrence** | 6.8001 | 0.4306 | 14.5971 | 0.072 |
| Region (Lyell Highway) | -4.8338 | -9.6526 | -1.2638 | 0.018* | **Region (Lyell Highway)** | -6.5884 | -12.8799 | 3.1019 | 0.156 |
|  |  |  |  |  |  |  |  |  |  |
|  | ***C. tasmaniensis*** | | |  |  | ***L. burrowsae*** | | |  |
|  | mean | low-95% CI | upper-95% CI | |  | mean | low-95% CI | upper-95% CI | |
| Site Nested (RE) | 16.58 | 0.000582 | 52.27 |  | **Site Nested (RE)** | 32.33 | 1.293 | 81.81 |  |
| Year (RE) | 2.897 | 0.000174 | 13.01 |  | **Year (RE)** | 1.073 | 0.00032 | 5.2 |  |
| site prop | 0.675582 | 0.001640 | 0.975847 |  | **site prop** | 0.915123 | 0.65232 | 0.987898 |  |
| year prop | 0.122340 | 0.000106 | 0.665992 |  | **year prop** | 0.030992 | <0.0001 | 0.254677 |  |
|  |  |  |  | pMCMC |  |  |  |  | pMCMC |
| (Intercept) | -0.7781 | -5.4507 | 3.7268 | 0.704 | **(Intercept)** | -47.4072 | -80.2938 | -18.5728 | <0.001* |
| Bd Status | 3.4552 | -0.8576 | 9.4374 | 0.164 | **Bd Status** | 1.2104 | -2.5989 | 5.6046 | 0.520 |
| Cs Occurrence | 0.7043 | -4.3634 | 5.6454 | 0.732 | **Le Occurrence** | 45.1027 | 18.1276 | 77.0248 | <0.001* |
| Region (Lyell Highway) | 5.4279 | 0.1624 | 12.0295 | 0.026* | **Region (Lyell Highway)** | 6.432 | -0.4612 | 14.8884 | 0.058 |
| Bd * Cs interaction | -1.2279 | -9.7792 | 6.7255 | 0.702 | **NA – no interaction** |  |  |  |  |
